# *Delphinium* as a model for development and evolution of complex zygomorphic flowers

**DOI:** 10.3389/fpls.2024.1453951

**Published:** 2024-08-19

**Authors:** Bharti Sharma, Mankirat Kaur Pandher, Ana Quetzali Alcaraz Echeveste, Rene Kenny Romo, Marianellie Bravo

**Affiliations:** Department of Biological Sciences, California State Polytechnic University, Pomona, CA, United States

**Keywords:** *Delphinium*, zygomorphy, synorganization, model system, evo-devo, petaloid spurs

## Abstract

The complex zygomorphic flowers of the early-diverging eudicot *Delphinium* provide an opportunity to explore intriguing evolutionary, developmental, and genetic questions. The dorsal perianth organs, consisting of a spurred sepal and the nectar-bearing spurred petal(s) in *Delphinium*, contribute to the dorso-ventralization and zygomorphic flower morphology. The seamless integration of the two or three dorsal petaloid spurred organs is considered a synorganization, and the resulting organ complex is referred to as a hyperorgan. The hyperorgan shows variability within the tribe due to variation in the number, size, and shape of the spurs. Research in recent decades within this tribe has enhanced our understanding of morphological evolution of flowers. More recently, functional studies using the RNAi approach of Virus-Induced Gene Silencing (VIGS) have unraveled interesting results highlighting the role of gene duplication in the functional diversification of organ identity and symmetry genes. Research in this early-diverging eudicot genus bridges the gaps in understanding the morphological innovations that are mostly studied in model grass and core eudicot clades. This first comprehensive review synthesizes eco-evo-devo research on *Delphinium*, developing a holistic understanding of recent advancements and establishing the genus as an exceptional model for addressing fundamental questions in developmental genetics, particularly in the evolution of complex flowers. This progress highlights *Delphinium’s* significant potential for future studies in this field.

## 1 Introduction

The flowering plants or angiosperms trace their evolutionary lineage back to around 140 million years ago (Mya) (Magallón et al., 2015; Sauquet et al., 2017, 2022). Considering the earth’s evolutionary history, angiosperms have diverged dramatically in this brief period. As a result, the existing +350,000 angiosperms display a stunning array of floral diversity (Endress, 2011; Christenhusz and Byng, 2016). This diversity provides an opportunity to understand complex evolutionary and genetic mechanisms that have sculpted novel and intriguing developmental innovations.

**Figure 1:**
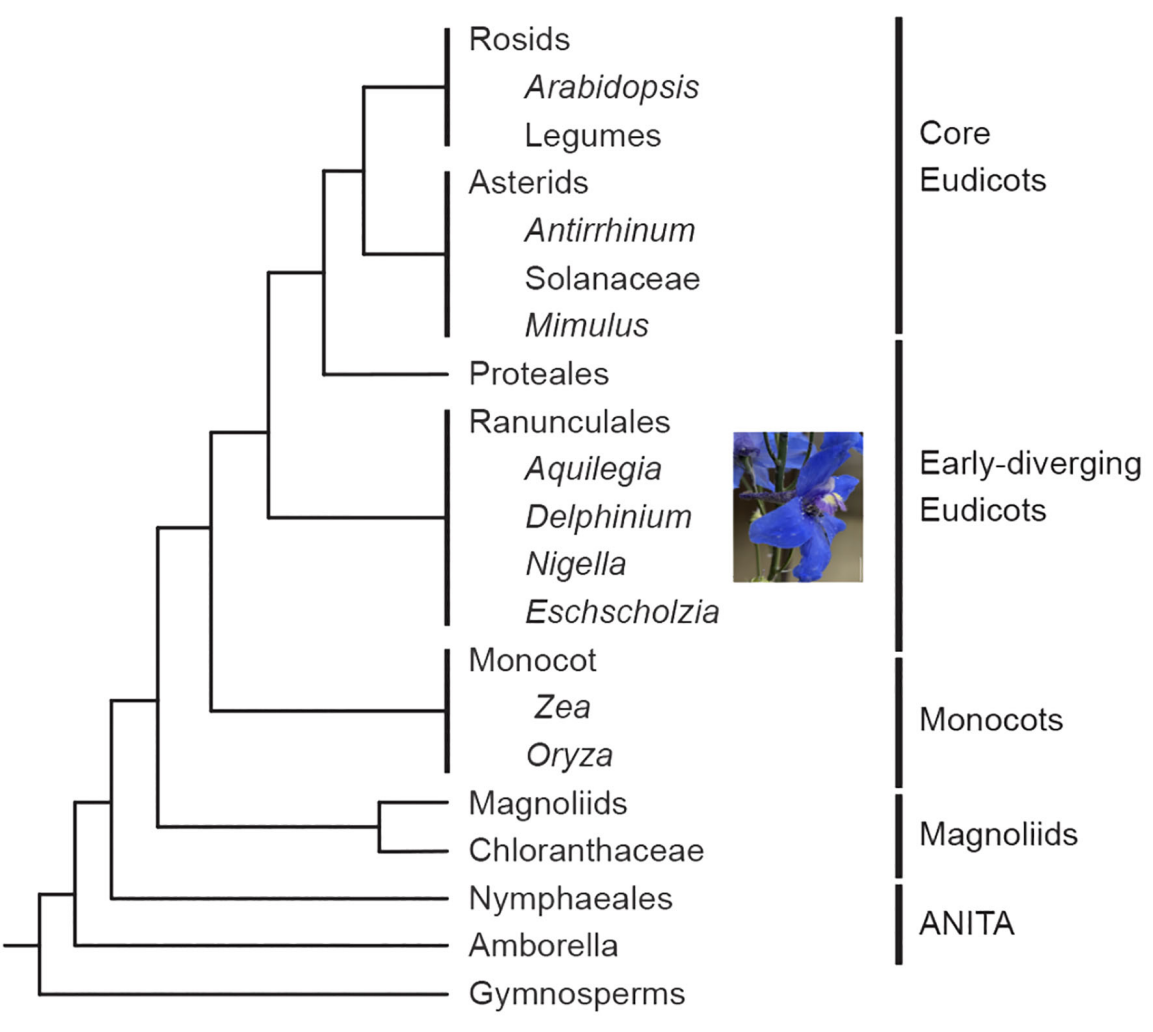
Simplified phylogeny of the angiosperms based on Moore et al. (2007) and Kramer and Hodges (2010) showing the position of *Delphinium* relative to other major model systems. Scale bar: 1 cm.

**Figure 2:**
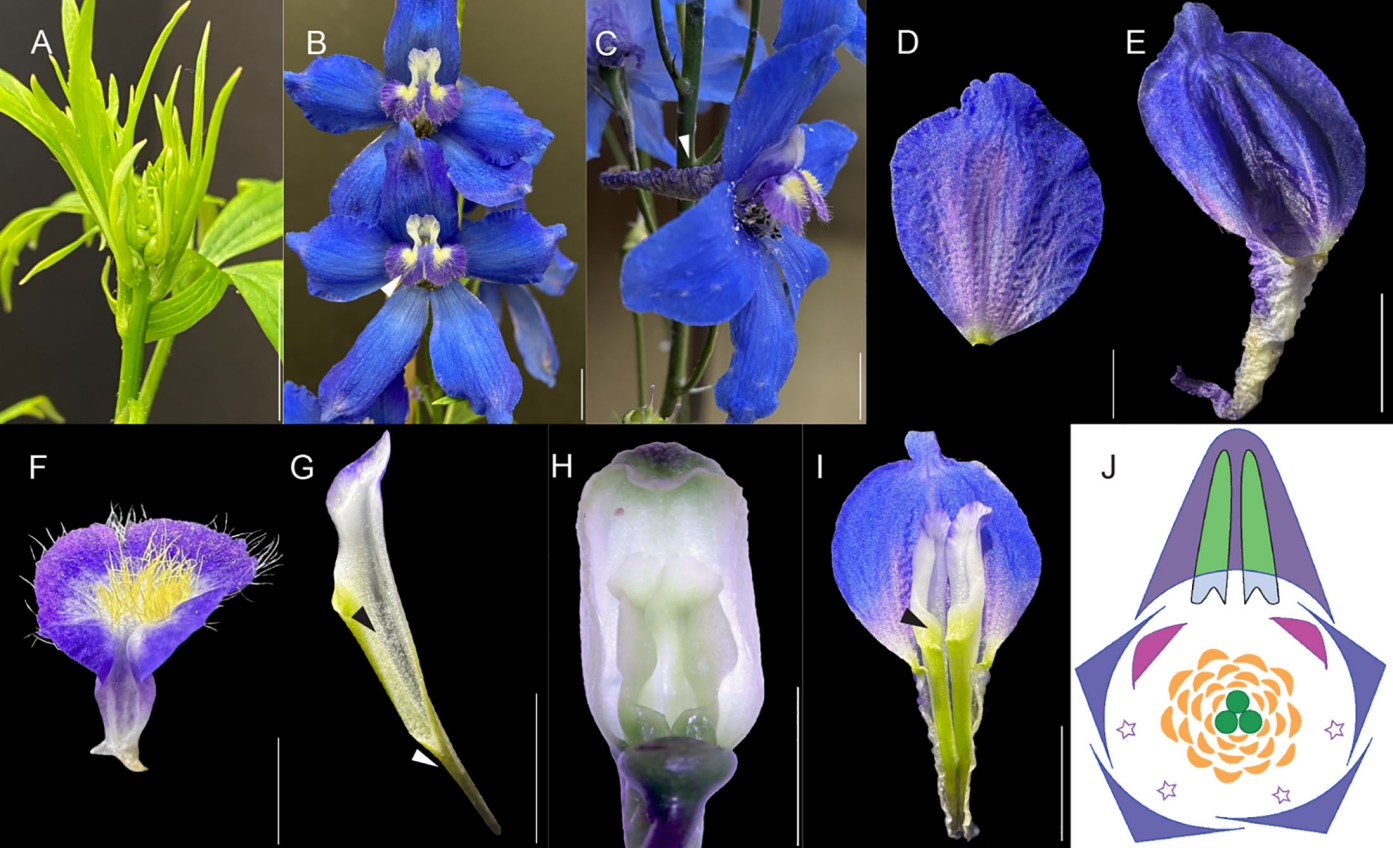
Photos of *Delphinium* x *belladonna “Bellamosum”* flower showing the arrangement of perianth organs, spur arrangement, and its floral organs appearance. **(A)** inflorescence, **(B)** front view with white arrow indicating trichome rich, non-spurred lateral petals, **(C)** side view with white arrow indicating sepal spur, **(D)** sepal, **(E)** spurred sepal, **(F)** non-spurred lateral petal, **(G)** spurred petal, black arrow pointing to the indented area, and white arrow pointing to three-dimensional cavity, **(H)** Spur-in-spur arrangement showing paired spurred petals residing within spurred sepal, **(I)** black arrow pointing to nectar-bearing spurred petal **(J)** floral diagram - in the dorsal region, there is a single-spurred sepal (purple), paired spurred petals (green spurs and light blue labium). Within the outer whorl, four sepals are present (blue-purple), and in the second whorl, reduced petals (stars), and two trichome-rich, non-spurred lateral petals (pink) are present. In the third whorl, stamens are present (yellow), and the fourth whorl contains carpels (green). Scale bars A-C, E, G, and I: 1 cm and D, F, and H: 0.5 cm.

Nestled within this diversity is the early-diverging eudicot order Ranunculales, which includes the genus *Delphinium*, noted for its zygomorphic floral morphology. Floral symmetry is predominantly classified into two types: radial symmetry or actinomorphy with more than two planes of symmetry, and bilateral symmetry or zygomorphy with one plane of symmetry. The Delphinieae tribe, to which *Delphinium* belongs, is comprised of approximately 650–750 species, representing ~25% of the Ranunculaceae family (**Figure 1**, Jabbour and Renner, 2012b; Novikoff and Jabbour, 2014; Espinosa et al., 2017). Zygomorphy has evolved several times in angiosperms but only once in Ranunculaceae, in the ancestors of the tribe Delphinieae (Jabbour et al., 2009a; Citerne et al., 2010; Jabbour and Renner, 2012b; reviewed by Hileman, 2014a; Reyes et al., 2016). The arrangement of perianth organs in *Delphinium* zygomorphic flowers represents a fascinating example of morphological diversification this makes the genus popular for horticultural purposes (**Figures 2A-C**, Jabbour et al., 2009b). In *Delphinium*, the elaborate perianth is composed of two distinct whorls, each playing a crucial role in the flower’s overall form and function. The two whorls of perianth organs in *Delphinium* are the sepals and petals, both are petaloid. Petaloidy is the term used to define organs attractive to pollinators, the display of petaloidy is not limited to perianth organs; showy bracts, leaves, and other organs can also be petaloid (Rasmussen et al., 2009; Sharma and Kramer, 2017).

The first whorl (W1) of perianth organs consists of four simple and flat petaloid sepals (**Figure 2D**, two lateral and two ventral) and one dorsal sepal that is spurred (Espinosa et al., 2021a). The asymmetrically elongated dorsal spurred sepal is particularly intriguing. It is noteworthy to mention that these spurs do not have nectaries. These spurs are petaloid, colored, and attractive (**Figures 2C, E**). Besides the aesthetic appeal of the flower, the petaloid sepals serve functional purposes in pollination and protecting reproductive organs (Macior, 1975). Spurs have independently evolved in Delphinieae relative to *Aquilegia*, which is also a member of the Ranunculaceae family (Delpeuch et al., 2022; Li et al., 2024a).

Within the second whorl (W2) are two lateral, showy petals that are trichome-rich in the center, often with a yellow spot and a pubescent texture (**Figures 2B, F**). These petals are not present in the genera *Aconitum* and *Gymnaconitum*, or in the subgenus *Consolida* of *Delphinium*. In addition to the showy petals are two (one in some species) dorsal, spurred petals (**Figures 2G, H**) that bear nectaries. These nectariferous petal(s) grow inside the hollow vicinity of the sepal spur whose three-dimensional morphology completely envelops the tubular spurs of the petals while the labium of the dorsal petals remains open to pollinators. Under the labium, the spur is indented, the dorsal ends of each petal spur, below the indented portion, form a completely closed three-dimensional cavity (Jabbour et al., 2021, **Figure 2G**) The spurred petals function in attracting and guiding pollinators, ensuring effective pollination (Jabbour and Renner, 2012b). The close arrangement of these petals, growing snugly into the pocket of the spurred sepal, is a remarkable adaptation. This spur(s)-in-spur is considered a synorganization (Chen et al., 2018), and the resulting structure is referred to as a hyperorgan (**Figures 2H, I**, Jabbour et al., 2021). Besides being visually striking, it has been speculated that this spur(s)-in-spur configuration serves a protective function, shielding the delicate nectaries from environmental stressors and protecting the integrity of the nectar, preventing leakage and adulteration (Chen et al., 2018). Paired and nectariferous petal spurs are only observed in Delphinieae in the Ranunculaceae family (Jabbour and Renner, 2012b).

The micromorphological features of the dorsal sepal spur (W1) and petal spurs (W2) are quite distinct (Jabbour and Renner, 2012b; Zhao et al., 2023). This complex arrangement of perianth organs in *Delphinium* flowers reflects a sophisticated interplay between genetic predisposition and environmental adaptation, leading to the development of structures that optimize pollination efficiency and hence contribute to the fitness of this tribe (Jabbour et al., 2009b). The analysis of these features in studies detailed in this review provides invaluable insights into the broader patterns of floral evolution and diversification among angiosperms.

This is the first comprehensive review that compiles the insights derived from various morphological, developmental, and genetic studies that elucidate floral morphology and organ identity in *Delphinium*. Through a focused exploration of biogeography, morphology, and molecular studies on perianth organization and symmetry, we develop a primer on the overall progress made in unraveling the evolutionary strategies that shape the distinctive flowers in *Delphinium*. Moreover, this review endeavors to articulate future research directions, positioning *Delphinium* as a key model system for understanding the complexities of flower development and the evolutionary marvel of zygomorphic flowers in the vast and diverse world of angiosperms.

## 2 Biogeography and genetic diversity

The *Delphinium* subgenus, in addition to three genera, namely *Staphisagria*, *Aconitum*, and *Gymnaconitum* collectively form the Delphinieae tribe (Jabbour and Renner, 2012a; Zalko et al., 2021). *Staphisagria* was raised to the level genus after the resurrection of the genus *Staphisagria* J. Hill (Jabbour and Renner, 2011b; Jabbour and Renner, 2012a). The evolutionary trajectory of this tribe is purported to have originated during the early Oligocene period, >32.3 million years ago (Jabbour and Renner, 2011a). The precise identification of their ancestral geographical region was rendered challenging by the unresolved status of their sister group of the tribe (Jabbour and Renner, 2012a). Recent studies suggest that the tribe Nigelleae is the sister group of Delphinieae (Lehtonen et al., 2016; Zhai et al., 2019).

**Figure 3:**
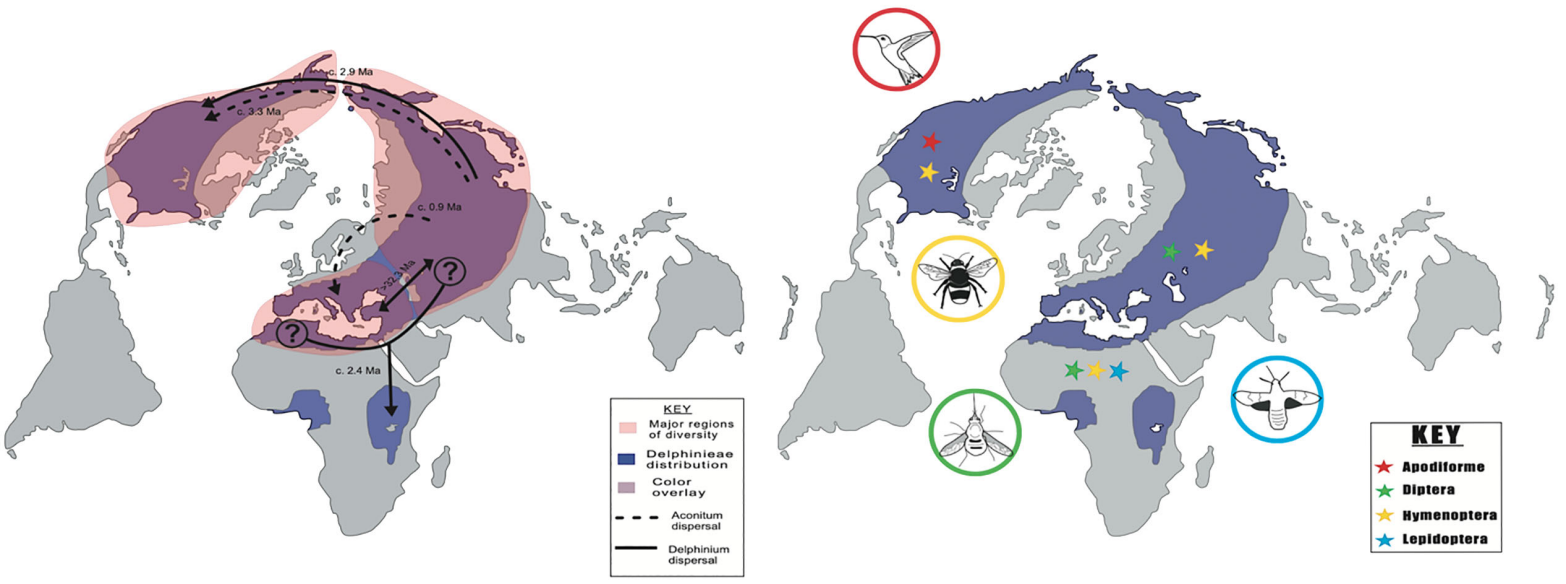
Regions of diversity, current day Delphinieae distribution and most common tribe pollinators. Adapted from Jabbour and Renner (2012a) (Left) Delphinieae’s major regions of diversity, the Mediterranean region, Asia (Eurasia), and North America. The sampling data did not include species from West Africa, origin source and genetic divergence was not inferred. *Aconitum:* dashed line. *Delphinium:* solid line. (Right) Present day Delphinieae distribution area represented by purple shading. Four most frequented pollinator orders among the tribe and their general geographic distribution; Hymenoptera (yellow), Lepidoptera (blue), Diptera (green), and Apodiformes (red).

The *Delphinium* genus is posited to have originated in East Asia during the late Oligocene around 24.27 Mya (Xiang et al., 2017). Subsequently, this genus underwent a notable evolutionary transition towards the perennial life cycle and a consequential expansion event in Asia during the late Miocene, approximately 9.7 Mya, facilitated by a phase of rapid diversification. Following this diversification across the Qinghai-Tibetan Plateau (QTP), *Delphinium* traversed into North America during the Pliocene, around 3 Mya, coinciding with the submergence of the Bering Strait (Brigham-Grette, 2001), and into East Africa (**Figure 3**, Jabbour and Renner, 2012a). Concomitantly, during this temporal epoch, *Delphinium* extended its geographical presence into the East African mountains, occurring between 0.7 and 4.4 Mya (Jabbour and Renner, 2012a; Chartier et al., 2016). This migration into East Africa was after the genus’s diversification in the Irano-Turanian region, coupled with its expansion into the Balkans and Italy between 7-8 MYA (**Figure 3**, Xiang et al., 2017).

Within Delphinieae, variation in ploidy levels has been observed. The online Delphinieae Chromosome Database (DCDB) http://www.delphinieae.online has recently been updated and is now online. This database provides information on chromosome number, karyological data, and estimated ploidy levels of members of the Delphinieae tribe. The information is based on published resources that have been deemed accurate by the authors. The database contains information on 425 species of this tribe (Bosch et al., 2023). The diploid number of chromosomes in Delphinieae ranges from 2n=12 to 2n=64. The most frequently observed karyotype among Delphinieae species is 2n=16 and 2n=32 (Bosch et al., 2023 http://www.delphinieae.online). However, chromosome numbers ranging from 2n=14 to 52 are also present (Bosch et al., 2023). *Aconitum* has the most variation in chromosome numbers (**Supplementary Table 1**). The authors suggest that this information can be particularly useful for cytotaxonomical databases and for systematic and evolutionary research (Bosch et al., 2023). Besides this, a recent study by Luo et al. (2023) reported a comparative karyomorphological analysis and genome size of five taxa from *Delphinium* sub genus *Anthriscifolium*. The diploid genome size ranged from 3.02–3.92 pg, while for tetrapolid it ranged from 6.04–6.60 pg (Luo et al., 2023, **Supplementary Table 2**). Additionally, the genome of *S. picta* is being sequenced, and expression atlases are being produced (The RanOmics group et al., 2024).

Chloroplast (cp) genomes are valuable tools for studying evolutionary relationships and have been widely used in reconstructing phylogenetic relationships (Soltis and Soltis, 1998; Li et al., 2021, 2024). In Delphinieae, the cp genome has been sequenced for various species in recent years and is now being deployed to study phylogenetic and phylogeographic relationships within the genus (Duan et al., 2020; Li et al., 2020; Park et al., 2020; Song et al., 2024).

## 3 Pollinators and pollination

Pollinators play a pivotal role in the reproductive success of plants. The relationship of plants with their pollinators has been the central focus of evolutionary studies. Changes in plant-pollinator relationships are often due to changes in morphology, including but not limited to color, shape, novel organs, or changes in nectar production or flowering time. Within the Delphinieae tribe, adaptation to insect pollination is observed. This might be an outcome of the floral morphology that features a floral parlor that not only attracts and guides the pollinators but also conceals the sugary nectar. Leppik (1964) defines parlor as a reception area for pollinators, in Delphinieae, the shared cavity in the hyperorgan is considered as a parlor. This unique morphology entices insects into repeatedly inserting and retracting their tongues or proboscises to access the nectar. These movements are implicated to maximize pollination and fit into the broader context of coevolutionary relationships that plants and pollinators share (Bosch et al., 1997). The existing studies have provided valuable insights into the interactions between the Delphinieae tribe and its pollinators. The observed pollinators within Delphinieae are bees (queen, bumblebees, and solitary in some cases), hummingbirds, hawkmoths, and the wind (**Figure 3**, Jabbour et al., 2009b; Jabbour and Renner, 2012b *).*

Amongst the insect pollinators, bee pollination is predominant, with more than 90% of Delphinieae species exhibiting this pollination mode. However, North American *Delphinium* species display variation. For instance, species such as *D. barbeyi, D. nuttallianum, D. tricorne*, in addition to bees rely on hummingbird-facilitated pollination (Jabbour and Renner, 2012b). In contrast, *D. cardinale*, and *D. nudicaule* rely exclusively on hummingbird-facilitated pollination. In East African species, *D. leroyi* hawkmoth pollination is observed (Johnson, 2008; Chartier et al., 2016). Bees and hawkmonth pollination have been observed in, *D. obcordatum* and *D. verdunense*, as well as in two species of *Staphisagria* subgenus (**Supplementary Table 3**, Jabbour and Renner, 2012a). In many Delphinieae species, effective pollinators are not specialized; however, bees pollinate the short-spurred species, the longest-spurred species are pollinated by hawkmoths, and medium-spurred varieties are pollinated by hummingbirds (Jabbour et al., 2009b). A similar pattern is also observed in *Aquilegia*, another genus within the Ranunculaceae that has evolved petal spurs independently (Hodges, 1997).

Within the *Staphisagria* species, the lowest activity of insects has been reported, which can be a result of imperfect herkogamy, leading to selfing (Bosch et al., 1997, 2001). The prevailing interferences suggest that Delphinieae has undergone very few pollinator switches (Jabbour and Renner, 2012b). The mean length of inner spurs ranges approximately from 4–30 mm in *Delphinium* and *Staphisagria* (Jabbour and Renner, 2012b). Bee-pollinated species in high-altitude southeast China, *D. tatsienense* and *D. oxycentrum*, and hawkmoth-pollinated species, *D. leroyi* in tropical Africa exhibit the longest spurs, 30, 34 and 37.5 mm respectively (Jabbour and Renner, 2012b). Bosch et al. report a clear correlation between altitudes and percentage of *Bombus* that visit the taxons growing in these altitudes (Bosch et al., 1997). Notably, no correlation between spur length and altitude was reported by Jabbour and Renner (2012b). It should be mentioned that Jabbour et al. (2021) suggest a re-assessment of relationship between the Delphinieae species and its pollinators. Such a re-assessment is necessary because traditionally outer spur lengths have been measured and reported in botanical and taxonomic treatments, although the nectar reward is provided by inner spurs (Jabbour and Renner, 2012b; Jabbour et al., 2021.

### 3.1 Nectaries and nectar secretion

A study by Antón and Kamińska (2015) analyzed the anatomy of floral spurs and the method of nectar exudation in four species of the Ranunculaceae family, including, *Aconitum lycoctonum* L., *Aquilegia vulgaris* L., *Consolida regalis* Gray, and *Delphinium elatum* L. These species share a general structural arrangement of nectaries, consisting of a single layer of internal epidermis, several layers of nectar-producing parenchyma cells, and underlying ground parenchyma. Despite this structural similarity, the method of nectar secretion varies among species. In *A. lycoctonum* and *A. vulgaris*, nectaries are located at the apices of the spurs, and nectar is produced through a holocrine method, where the epidermal cell wall erupts, resulting in nectar containing disrupted cell organelles such as mitochondria and nuclei. In contrast, in *D. elatum* and *C. regalis*, nectaries are located along the floor of the spur, with nectar exuding through microchannels into the cuticle; the secretory tissue is positioned along the ventral surface of the spur (Antoń and Kamińska, 2015).

Additionally, the study highlights the relationship between nectary spur length and pollinator type. The relatively long nectar spurs of the studied species are associated with long-tongued bumblebees, their primary pollinators (Jabbour and Renner, 2012b; Antón and Kamińska, 2015).

## 4 Diversity in floral organ morphology and synorganization in Delphinieae

**Figure 4:**
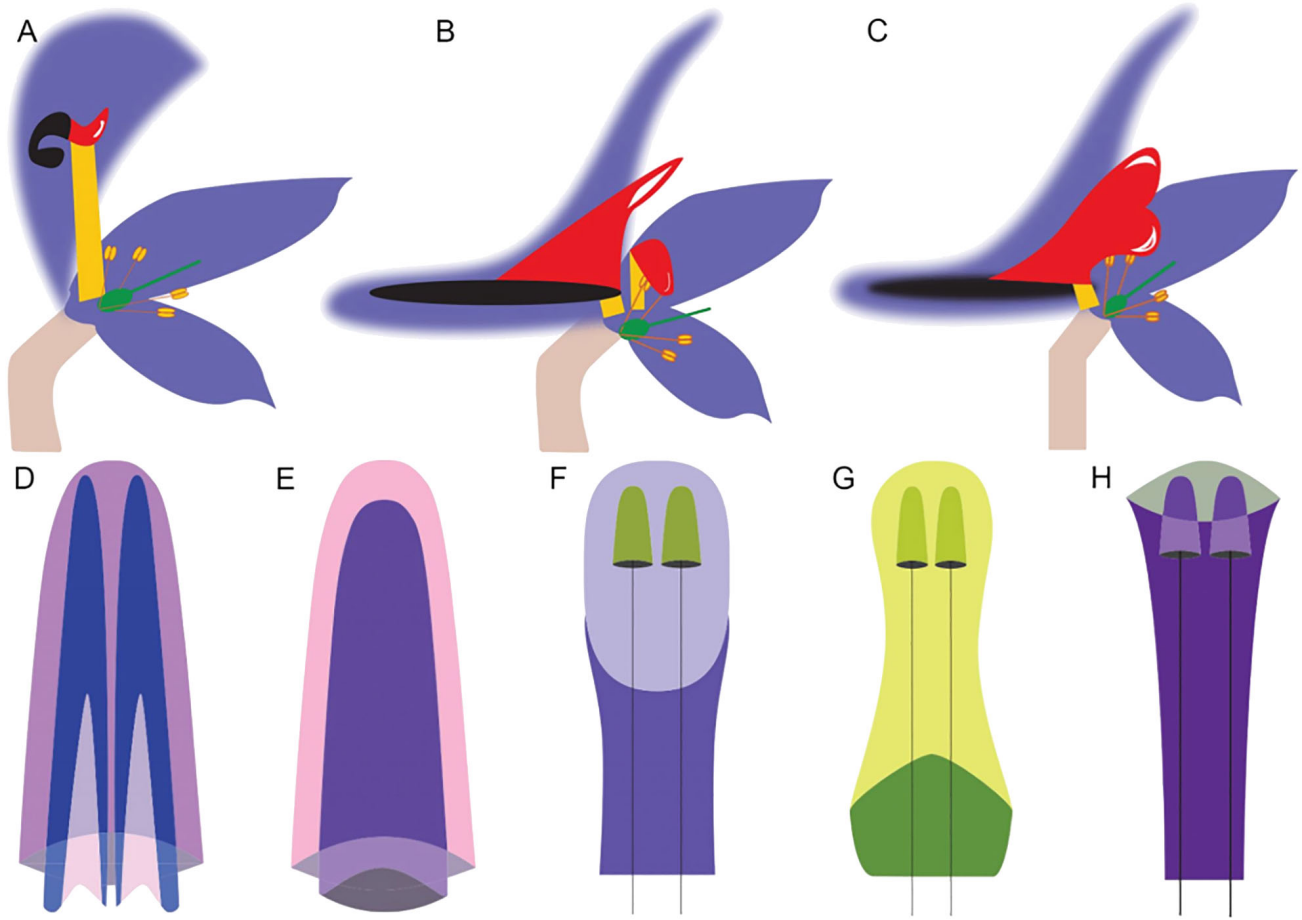
Differences in the morphology of the hyperorgan of the *Delphinieae* tribe. **(A-C)** (Adapted from Jabbour and Renner (2012b); Jabbour et al. (2021)), schematic longitudinal sections of perianth organization, Hooded type, Spurred type, and Spurred type with fused W2 organs, respectively. W1 organs are shown in purple, W2 organs stalk, labium, and spur are shown in yellow, red, and black, respectively. Half organs are drawn with faded lines. Gynoecium and androecium are represented by the green structure and protruding stamens. The number of carpels and stamens vary depending on the species. **(D)**, Spurred type hyperorgan with two spurs inside a third found in *Staphisagria*, *Delphinastrum*, *Oligophyllon*, *Anthriscifolium*, and *Delphinium*. **(E)**, Spurred-type with fused W2 organ with one spur inside a second found in *Consolida*. **(F, G)**, Hooded type hyperorgan with two short spurs on long pedicels enveloped by a helmet or cap shaped sepal found in *Aconitum*. **(H)** Hooded type hyperorgan with two short spurs on long pedicels covered by a portruding sepal found in *Gymnaconitum.*.

Within the Delphinieae tribe, flowers exhibit remarkable morphological diversity, yet all share a common structural plan. As mentioned above, the calyx is petaloid and elaborate with a dorsal sepal sculpted into a pocket or hood (spur), varying in length and shape across the tribe (Chen et al., 2018). The dorsal enclosed spurred petal(s) comprise three components: a stalk, spur, and labium, with variations in number and their proportions across different Delphinieae clades (Jabbour and Renner, 2012b; Chen et al., 2018). Because of the variation in morphology (of both sepal and petal(s) spur) and in the number of petals, the overall three-dimensional structure of the hyperorgan itself shows variation, as explained with specific examples below (**Figure 4**). In many species of Delphinieae, ventral and lateral petals have been observed. Interestingly, the development of ventral petals (shown as stars in **Figure 2J**) is arrested at an early developmental stage. Their number varies from 4–8, and these petals have been referred to as reduced petals in the literature. There is one report that describes the stage at which the development is arrested and triangular reduced and aborted non-spurred petals are observed (Zalko et al., 2021). Besides, trichome-rich, showy, non-spurred lateral petals are observed in many species, including those in the genus *Staphisagria* and the subgenera *Delphinium*, *Anthriscifolium*, *Delphinastrum*, and *Oligophyllon* of the genus *Delphinium* (Zalko et al., 2021).

The morphological diversity of the hyperorgan of the tribe Delphinieae has been categorized into three types based on perianth organization: hooded-type, spurred-type, and spurred-type with fused W2 organs (**Figures 4A-C**, Jabbour and Renner, 2012b). Within the hooded-type perianths, further variations are noted, such as the dorsal sepal appearing as a helmet or as a cap, protruding and elaborating over the curved nectar-bearing petals (**Figures 4D-H**). Helmet, cap-type, protruding short spurs carried on a long pedicle with a stalk much longer than the labium can be found in the genus *Aconitum.* (Jabbour et al., 2021). The *Delphinium* subgenus and *Staphisagria* exhibit spurred-type hyperorgan morphology where the two most dorsal petals are spurred and encased within a dorsal spurred sepal. The spurred type with fused W2 organs is observed in *Consolida* which is a subgenus of *Delphinium* (DuPasquier et al., 2021; Espinosa et al., 2021b). Similarly to *Aconitum*, reduced primordia also evolved in subgenus *Consolida* alongside the fusion of the W2 dorsal primordia as well as the development of basal wings on the sides of the W2 adult organs (Jabbour and Renner, 2012b).

## 5 Petals and their molecular basis in *Delphinium* and *s*tanding questions on petaloidy of sepals

The evolution of petals stands as one of the major factors underlying the fitness and perhaps rapid radiation and diversification of angiosperms (Drea et al., 2007; Whittall and Hodges, 2007). Within the Ranunculaceae family, the morphological diversity in perianth organs is notable. The floral diversity is especially exemplified by nectaries and elaborate floral perianth organs. Most commonly, a bipartite perianth that is composed of sepals in the outer whorl and petals in the second whorl is observed in this family (Kosuge, 1994; Jensen and Kadereit, 1995). However, the family is also characterized by a perianth that includes elaborate spurs, asepalous and apetalous taxa, and organs with ambiguous identities that can represent either sepals or petals (Rasmussen et al., 2009). Such diversity is specifically noted in genera such as *Nigella* (forked petals with pseudonectaries), *Aquilegia* (nectar-bearing petal spurs, petaloid sepals), and in recent years more extensively discussed in *Delphinium.* Within the tribe Delphinieae, both whorls of perianth organs are petaloid, and the complex perianth has spurred and non-spurred forms of sepals and petals. The hyperorgan makes the flowers elaborate. This kind of diversity between and within the whorls begs obvious explanation from a developmental genetics perspective. Simply, the obvious question that entreats exploration is - What genetic programs contribute to the establishment of distinct petaloid perianth organs in *Delphinium*? Do the nectar and non-nectar-bearing spurs share a common or distinct genetic organ identity program?

The genetic bases of floral organ identity establishment were put forth by the “ABC”, “ABCE” and “Quartet” models (Bowman et al., 1991; Coen and Meyerowitz, 1991; Honma and Goto, 2001; Theissen and Saedler, 2001). As proposed by these models based on functional studies in core eudicots, the expression of A+E is required for sepal identity, A+B+E for the establishment of petal identity. Conservation of A function outside core eudicots is not observed (Litt, 2007).

Gene duplications within ABCE genes has resulted in multiple paralogs. In the Ranunculaceae family, two such duplication events have occurred (~70–120 Mya) within the B class *APETALA3* (*AP3*) gene lineage that have resulted in *AP3-1*, *AP3-2* and *AP3-3* lineages (Kramer et al., 2004; Rasmussen et al., 2009; Zhao et al., 2023). The homologs of these are retained in *Delphinium*, *DeajAP3-1*, *DeajAP3-2*, and *DeajAP3-3*. Similarly, in *Delphinium* the other B gene homolog, *PISTILLATA* (*PI*) has two copies *DeajPI1* and *Deaj PI2.* Three homologs of the E class gene, *SEPALLATA (SEP) Deaj Sep1*, *DeajSEP-2*, and *DeajSEP-3* have also been reported (Zhao et al., 2023).

A recent study by Zhao et al. (2023) reports the function of B-class genes based on the functional study conducted using VIGS. How the functional roles of these duplicate genes have evolved and how the resulting genetic networks have contributed to sculpting the complex zygomorphic flowers and the diversification of the tribe Delphinieae, is something that needs to be explored meticulously with functional genetics.

The expression of B genes in developing *Delphinium* flowers shows some conserved expression patterns but also highlights differentiated expression among paralogs (**Supplementary Table 4**). The expression patterns of D*eajAP3-1* and *DeajAP3-2* are broad, but both are strongly expressed in stamens*. DeajAP3-3* is highly expressed in the dorsal petal spurs. Both *PI* paralogs are differentially expressed. *DeajPI1* is highly expressed across all developmental stages and all floral organs except in developed carpels*. DeajPI2* expression is most prominent in stamens at later developmental stages but in early developmental stages across all organs, it shows a milder expression. The E class *SEP* paralogs also show differential expression, *DeajSEP3* gene is shown to be expressed across all organs and developmental stages, while the expression of *SEP1* and *2* homologs is broad and weak. *DeajSEP2* is not expressed in matured stage (S12) dorsal nectiferous petals and stamens (Zhao et al., 2023).

**Figure 5:**
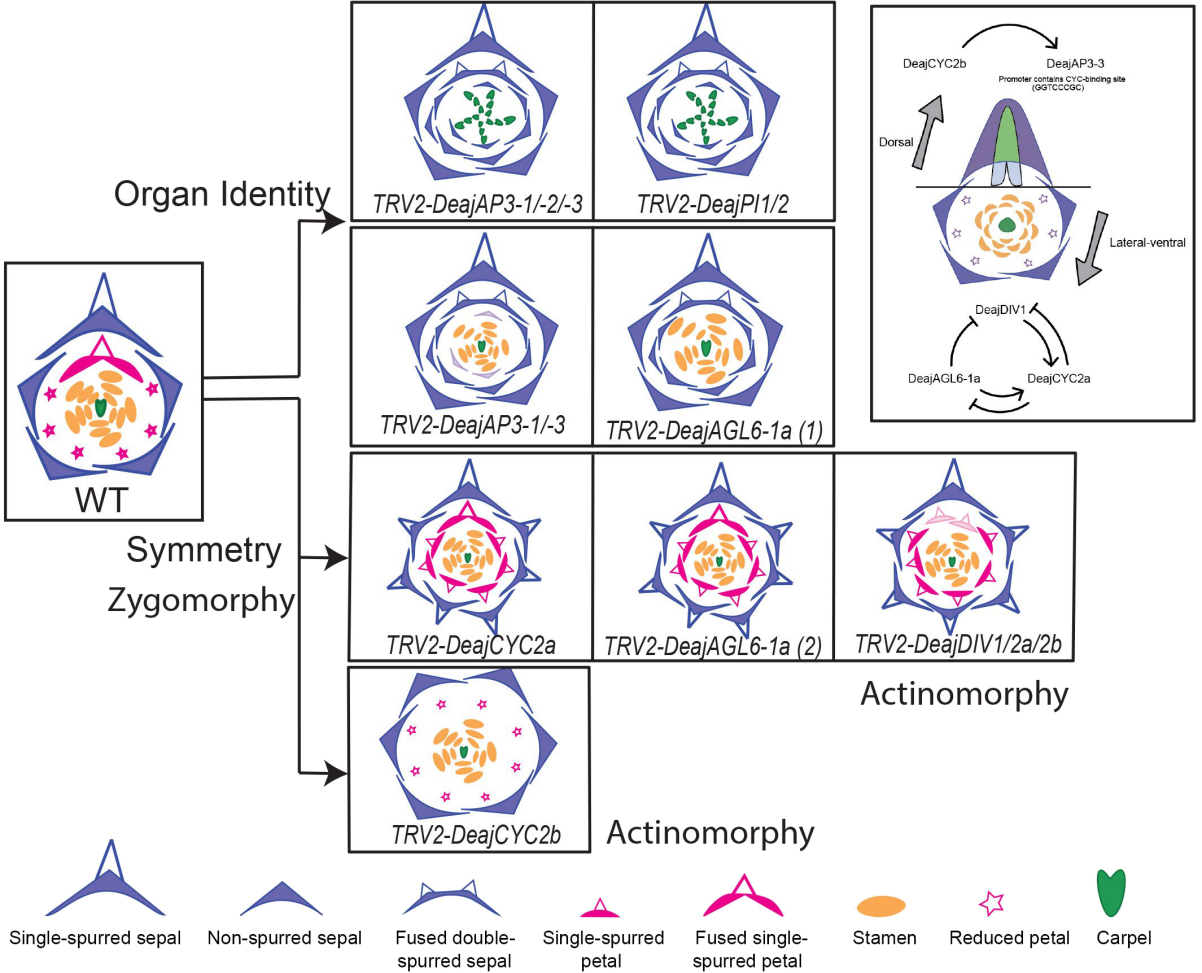
Relationship between floral organ identity and symmetry genes as observed in *D. ajacis*. Knockdown phenotypes of different floral organ identity and symmetry genes are compared to observe patterns. Gene knockdown that produces similar phenotype are placed in the same row. Through this comparison, floral organ identity genes and symmetry genes can be clearly separated, and interestingly, the role of *DeajAGL6-1a* in both organ identity and symmetry can be observed here. To the right, regulatory links between floral symmetry and organ identity genes are shown in the dorsal and lateral-ventral region. Arrows indicate positive regulation, while blunt arrows represent negative regulation; the dotted line separates dorsal and lateral-ventral regions. Floral organ diagrams adapted from Zhao et al., 2023.

The availability of genetic resources, specifically a functional tool such as VIGS, as recently reported by Zhao et al., 2023, has made it possible to unravel the functional role of important genes that orchestrate the establishment of floral organ identity in *Delphinium ajacis* (*Deaj*). Functional knockdown of the *AP3-3* homolog in *Delphinium ajacis* (*DeajAP3-3*), a species that exhibits a fused single-spurred dorsal petal and lacks lateral non-spurred petals, results in the homeotic conversion of spurred nectary bearing petal into fused double spurred sepal. The knocking down of *DeajAP3-3* and *DeajAP3-1* together resulted in the reduced petals homeotically converted into sepals, exhibiting more than two outer whorls of sepals followed by the stamens and carpels. The dorsal petal homeotically transforms into a fused, double-spurred sepal. The phenotypes obtained by gene knockdown of all three homologs of *AP3-3* (TRV2-*DeajANS*-*DeajAP3-1*, and *DeajAP3-2, DeajAP-3*) are classic B knockout phenotypes, with outer whorls consisting of sepals and inner whorls comprising carpels. Also, the dorsal single-spurred petal homeotically transforms into a fused double-spurred sepal (**Figure 5**). Interestingly, the reduced petals are homeotically transformed into sepals. The exact phenotype is also obtained when both *PI* homologs (TRV2-*DeajANS*- *DeajPI1 and DeajPI1)* are knocked down together (**Figure 5**). These results highlight that AP3-3 and PI proteins are in an indispensable partnership and they function together as obligate heterodimers. *AP3-1* and *AP3-2* have a role in stamen identity whereas *AP3-3* and *AP3-1* together have functional overlap in regulating petal identity.

It is important to note that in B gene knockdowns, the dorsal petal spur’s identity changes from fused single-spurred petals into fused double-spurred sepals through homeotic transformation. Although the spur identity changes, the 3D spur development and outgrowth still occur due to complete homeosis, however, it is interesting that the homeotic organ has two spurs with fused labium but unfused spur outgrowth (**Figure 5**). The dorsal spur outgrowth in *Delphinium* also contributes to zygomorphy, hence it can be implicated that the organ identity program of dorsal spurs is downstream of the zygomorphy program. In actinomorphic *Aquilegia*, spur outgrowth is lost with the downregulation of *AP3-3 or PI* homologs (Kramer et al., 2007; Sharma et al., 2011), however, this is complete homeosis as sepals are not spurred in *Aquilegia*. This further implies that the evolution of spur in dorsal petaloid organs in *Delphinium* might have caused the shift from actinomorphy to zygomorphy. As a future avenue, it will be interesting to explore how zygomorphy contributes to spur formation in both petaloid whorls.

As mentioned above, lateral petals are not present in *D. ajacis* but recent studies by Zhang et al. (2024a) have used *D. anthriscifolium*, which exhibits lateral petals, as a model system to provide new insights. In this species, the *DeanAP3-3* knockdown resulted in all petals, including dorsal spurred (2), lateral (2), and reduced petals (4), being homeotically transformed into flat non-spurred sepals. This result contrasts with the findings of Zhao et al. (2023), where the dorsal petal was homeotically transformed into fused double-spurred sepals, and the reduced petal identity remained unaffected. The discrepancy could be due to a stronger gene silencing effect in the Zhang et al. (2024a) study. Alternatively, as suggested by Zhang et al. (2024a), there may be interspecific differences in the regulatory mechanisms of the *AP3-3* homolog. In *D. ajacis*, the *AP3-3* homolog may specifically regulate dorsal petal identity, while in *D. anthriscifolium*, it may control the identity of dorsal, lateral, and reduced petals (Zhao et al., 2023; Zhang et al., 2024a). In another study by Zhang et al. (2024b), the role of the class I HD-Zip transcription factor *LATERAL MERISTEM IDENTITY I* (*DeanLMI1*) gene homolog in petal asymmetric development was demonstrated using VIGS.

## 6 Developmental and genetic bases of zygomorphy in Delphinieae and the crossroads where symmetry and *MADS-box* genes intersect

Zygomorphy is a derived state in angiosperms and is often associated with pollinator specialization (Ronse De Craene et al., 2003; Zhang et al., 2013). The ancestral state for Ranunculaceae flowers is spiral phyllotaxy and an open floral ground plan, typically associated with actinomorphy. The transition from the ancestral state of actinomorphy to the derived zygomorphy happened once in Ranunculaceae in the ancestor of the Delphinieae tribe that further diverged into *Staphisagria, Aconitum*, *Gymnaconitum*, and *Delphinium* (Zalko et al., 2021). Generally, zygomorphy is associated with whorled phyllotaxy, however, in Delphiniae the floral organs are arranged on an ontogenic spiral (Payer, 1857). In developmental studies conducted on *D. staphisagria* and *D. grandiflorum*, the zygomorphy is established in a later developmental stage that coincides with the dorsal sepals and petal spur development (Jabbour et al., 2009b). The organogenesis process is predominantly centripetal the sepals develop first followed by petals, stamens, and finally carpels (Schöffel, 1932).

Reported by Jabbour et al. (2009b), the majority of the development and organ identity establishment happens in *Delphinium* in the first quarter of development, the developmental scale in this study was based on the base width of the floral bud to an adult flower. However, it is important to note that after petal primordia initiation, petal development is delayed and reinitiated later; it is at this time when the dorsal petals acquire the three-dimensional spur shape. The hood/helmet/spur in sepals develops after the petal spur. The establishment of zygomorphy happens when the bud is 2mm (Kosuge and Tamura, 1988; Jabbour et al., 2009b) in *D. grandiflorum;* however, a delay is reported in *D. staphisagria*. These developmental studies implicate that zygomorphy establishment is correlated with the 3D growth of the spur(s) in petals and subsequently in sepals.

Gene duplication within organ identity and symmetry genes has facilitated novel functions of the duplicated homologs, driving diversification and evolutionary radiation (Kramer and Zimmer, 2006; Sharma et al., 2014). These phenomena contribute significantly to the phenotypic complexity and adaptability. The *CYCLOIDEA* (*CYC*) and *DIVARICATA* (*DIV*) are integral to the regulation of floral symmetry and development in flowering plants. *CYC*, a TCP transcription factor, plays a critical role in establishing bilateral symmetry (zygomorphy) by regulating the development of dorsal petals (Zhang et al., 2010; Howarth et al., 2011; Zhang et al., 2013). *DIV*, another key player in floral symmetry, often works in conjunction with other genes to influence petal development and symmetry (Hileman, 2014b; Madrigal et al., 2019).

Within the Ranunculaceae family, there are two *CYC* clades, *RanaCyL1* and *RanaCyL2* (Jabbour et al., 2014). Delphinieae-specific duplication events have further resulted in two paralogs of each, *DelCYC1a*, *DelCYC1b*, *DelCYC2a*, and *DelCYC2b* (Zhao et al., 2023). In a recent study, Zhao et al. (2023) investigated the expression and function of homologs of *CYCLOIDEA, and DIVARICATA*. The *DeajCYC1a* homolog was expressed in ventral sepals only at stage 12 (**Supplementary Table 4**). This gene is more broadly expressed in *D. anthriscifolium* and *S. picta*, two other members of the Delphinieae tribe (Zhao et al., 2023). Notably, these species have lateral petals, that are absent in *D. ajacis*, and have only 4 reduced petals compared to six in *D. ajacis*. It is tempting to speculate the role of the *CYC1* homolog in regulating lateral petal development. No expression data on *DeajCYC1b* was reported in this study.

Furthermore, *DeajCYC2a* is not expressed in floral organs across any developmental stages (1, 2, 4, 9, 12, 16)*. DeajCYC2b* was shown to be moderate to highly and broadly expressed in the dorsal regions of sepals and petals, particularly in stages 4 and 6 (highly), and in the spurred petals and sepals of stages 9, and 12 (moderately, **Supplementary Table 4**). *DeajDIV1* has broader expression patterns, with high expression in stages 9 and 12, especially in all petaloid organs (Zhao et al., 2023). *DeajDIV2a* also have a broader expression pattern, with high expression in stage 9 dorsal and ventral petals. *DeajDIV2b’s* expression is low and is only noticed in floral organs of stages 9 and 12 (**Supplementary Table 4**, Zhao et al., 2023).

Functional analyses were conducted on all *CYC2* homologs and *DIV* homologs using VIGS (**Figure 5**, Zhao et al., 2023). A change in symmetry from zygomorphy towards actinomorphy is observed in all knockdowns, with *TRV2-DeajCYC2b* phenotypes exhibiting almost perfect actinomorphy. *DeajCYC2b* is specifically implicated in regulating dorsal spur development (**Supplementary Table 4**, Zhao et al., 2023). Silenced flowers exhibit six sepals and no spur growth is observed in dorsal sepals (**Figure 5**). The fused dorsal petals are transformed into a reduced petal and no spur formation is seen. This is different from B gene silencing where dorsal fused petals homeotically transform into fused double spurred sepals.

The silenced *DeajCYC2a* flowers contained mostly six sepals with spurs in the W1. The W2 petal phenotypes were also striking, with silenced flowers having seven spurred petals, including the dorsal fused petal with spur, and six other petals with spurs in place of reduced petals (**Figure 5**). The phenotype obtained suggests that *DeajCYC2a* may be expressed at a very low level, which was not detected in the expression studies. Alternatively, *DeajCYC2a* may have been transiently expressed during a specific developmental stage that was not captured in the expression data. The *DeajDIV1*/*2a*/*2b* knockdown phenotypes were quite similar to *DeajCYC2a*, with the difference that W2 dorsal petals are not fused in strongly silenced flowers. These phenotypes implicate a role of *DeajCYC2a* and *DeajDIV* homologs in the establishment of lateral and ventral identity of flowers by repressing the development of reduced petals and spurs in sepals (**Supplementary Table 4**, Zhao et al., 2023).

The *DeajCYC2* homologs underwent asymmetrical evolution, *DeajCYC2a* have experienced deletions and diverged markedly. Interestingly, in the expression studies presented by Zhao et al. (2023), *DeajCYC2a* is not expressed in floral organs of three different stages (9,12,16) in three *Delphinium* species (*D. ajacis*, *D. anthriscifolium* and *S. picta*, Zhao et al., 2023). However, its downregulation results in shifting the symmetry towards actinomorphy with all sepals that are spurred including extra spurred sepal(s), additionally all reduced petals homeotically transform into spurred petals (**Figure 5**). Based on functional data obtained from silencing *CYC2* homologs, this study inferred a plausible hypothesis that the ancestor of Delphinieae might have been actinomorphic with spurs in all petaloid organs, referred as all-spur-first hypothesis.

The expansive study by Zhao et al. (2023) also reported various regulatory pathways that link the interactions of floral organ identity and floral symmetry genes. One compelling piece of evidence was that the promotor region of *D. ajacis AP3-3* (*DeajAP3-3*) contains a CYC-binding site, GGTCCCGC, and the expression of *DeajCYC2b* precedes *DeajAP3-3* (**Figure 5**). This finding suggests *DeajCYC2b* can possibly act as a positive regulator for the *AP3-3* gene. Another intriguing finding of the study by Zhao et al. (2023) was the role of *AGL6* gene homologs in regulating spur development. *AGL6* are members of the type II MADS-box gene family (Dreni and Zhang, 2016). In *Delphinium* there are two *AGL6* homologs, *DeajAGL61a* and *DeajAGL61b*, both are broadly expressed across all floral organs (**Supplementary Table 4**). The resultant phenotype of downregulating *TRV2-DeajAGL61a* resembled that of *TRV2*-*DeajCYC2a* and to some extent of TRV2 *DeajDIV1/2a/2b* (**Figure 5**). As mentioned above, in the silenced flowers, the symmetry shifts towards actinomorphy, all sepals in W1 and petals in W2 were spurred, resulting in multiple spur-in-spur structures. Similar to *CYC2a* and *DIV* homologs, the reduced petals were homeotically transformed into spurred petals, the dorsal petal was fused and single-spurred. Analysis of the knockdown phenotypes of *AGL6* homolog implicated complex interactions between *AGL6*, *CYC*, and *DIV* homologs. Forming a feedback loop directly or indirectly the plausible hypothesis is, *AGL61a* and *DIV1* homologs promote the expression of *CYC2a* while *CYC2a* represses both. Additionally, *AGL6* represses *DIV1*. This study not only revealed the role of *AGL6* homolog in repressing spur formation but also in lateral and ventral petal development (Zhao et al., 2023). The exact functioning of how this feedback loop works needs further exploration.

## 7 Summary

Within angiosperms, the transitions from actinomorphy to zygomorphy have occurred several times independently. In the case of Delphinieae, this transition is also accompanied by the evolution of the spur(s)-in-spur synorganized hyperorgan. Variability in the size, number, and morphology of spurred organs contributes to the diversity in hyperorgan architecture observed across the tribe. As Delphinieae expanded geographically from East Asia to North America and Africa, their life cycles evolved from facultative annuals/biennials to perennials. Although bees are the primary pollinators across the tribe, some species are also pollinated by hawkmoths and hummingbirds.

Recent genetic and functional studies have revealed the co-option of *CYC*, and *DIV* homologs in regulating floral symmetry. Gene duplications in *CYC* genes, MADS-box organ identity, and *AGL6* genes have resulted in the evolution of complex morphology of *Delphinium* flowers. Particularly, the role of *DeajAGL61a* gene homologs in suppressing spur development and the possibility of its genetic interactions with *DeajCYC2a* in regulating floral symmetry have been intriguing findings.

Overall, this early-diverging eudicot genus provides a unique opportunity to understand the evolutionary and genetic mechanisms underlying zygomorphy and complex flower evolution. This review aims to comprehensively highlight the novelty and potential of *Delphinium* as a model system for evo-devo studies.

## 8 Future directions

Published work related to flower ecology and evo-devo in Delphinieae has set an excellent foundation for understanding the evolutionary and developmental processes underlying floral diversity. This work has underscored the opportunities for delving deeper into and using the morphological innovations in these complex flowers as the raw material to study the evolution of functional traits. Below, we highlight some key questions that can be addressed in future research.

- Resolving the Delphinieae species-level phylogeny to clarify the unresolved questions related to the origination of the tribe. How has the biogeographical expansion affected the morphology and the pollination syndrome in *Delphinium* begs more attention.
- A compelling question related to floral morphology is elucidating the genetic networks that control distinct petaloidy of spurred and non-spurred petals and sepals and understanding how zygomorphy regulates or modulates spur development in sepals and petals.
- Sequencing *Delphinium’s* genome will facilitate comparative genomic and transcriptomic studies. Given the size of the genome, this is a major undertaking, so an alternative approach would be to sequence gene-rich regions.
- Exploration of the biophysical and cell division aspects that sculpt the spur(s)-in-spur three-dimensional growth of the dorsal hyperorgan will help understand the phenomenon underlying complex three-dimensional organ growth.
- An intriguing avenue for research lies in understanding the evolution and rewiring of genetic pathways that underpin annual, biennial, and perennial lifestyles in the tribe Delphinieae. Comparative -omics studies in extant species in each life cycle group can yield a wealth of knowledge that can inform targeted crop improvements, thereby advancing agricultural sustainability and productivity.
