# Supplementary material for: Delphinium as a model for development and evolution of complex zygomorphic flowers
Source: Front Plant Sci. 2024 Aug 19;15:1453951. doi: 10.3389/fpls.2024.1453951 (PMC11366623; doi:10.3389/fpls.2024.1453951)
Supplement: Supplementary file 1 [file DataSheet1.docx]

Supplementary Material

*Delphinium* as a Model for Development and Evolution of Complex Zygomorphic Flowers

Bharti Sharma^1*^, Mankirat Kaur Pandher^1^, Ana Quetzali Alcaraz Echeveste^1^, Rene Kenny Romo^1^, Marianellie Bravo ^1^

*** Correspondence:** Corresponding Author: bsharma@cpp.edu

# Supplementary Tables

**Supplementary Table 1:** Chromosome number in the tribe Delphinieae obtained from Bosch et al., (2023), DCDB data.

(* uncommon)

| Delphinieae | **Basic Number** | **Chromosome Number (2n)** |
| --- | --- | --- |
| *Aconitum* | 6*, 8, 9*, 10*, 13* | 12,16,17,18, 20, 24, 26, 28, 30, 32, 34, 40, 46, 48, 52, 64 |
| *Gymnaconitum* | 8 | 16 |
| *Delphinium* | 8, 9*,10* | 16,18, 20, 24, 32, 48 |
| *Consolida* | 7*, 8, 9, 10* | 14,16,18, 20, 24 |
| *Aconitella* | 6*, 8, 9* | 12,16,18 |

**Supplementary Table 2:** Summary of chromosome number and genome size of *Delphinium* subgenus *Anthriscifolium*

| Taxa | Chromosome number | Ploidy | 2C (pg) | 1Cx (pg) | Source |
| --- | --- | --- | --- | --- | --- |
| *D. anthriscifolium* var.  *anthriscifolium* | 32 | 4x | 6.27  [avg] | 1.57  [avg] | Luo et al. 2023 |
| *D. anthriscifolium* var.  *savatieri* | 16 | 2x | 3.36  [avg] | 1.68  [avg] | Luo et al. 2023 |
| *D. anthriscifolium* var.  *majus* | 16 | 2x | 3.82  [avg] | 1.91  [avg] | Luo et al. 2023 |
| *D. ecalcaratum* | 16 | 2x | 3.03  [avg] | 1.52  [avg] | Luo et al. 2023 |
| *D. callichromum* | 16 | 2x | 3.1  [avg] | 1.55  [avg] | Luo et al. 2023 |

Adapted from Luo et al. 2023 (DOI: 10.3897/phytokeys.234.108841)

The chromosome number, ploidy, 2C, and 1Cx values for the five species listed above were averaged from a larger data set. The data included eight populations of *D. anthriscifolium* var.  *anthriscifolium*, seven of *D. anthriscifolium* var.  *savatieri*, three of *D. anthriscifolium* var.  *majus*, two of *D. ecalcaratum*, and two of *D. callichromum* (Luo et al., 2023)*.*

**Supplementary Table 3.** Recorded pollinators and phenology for taxa of the Delphinieae tribe

Summary of pollinator orders, continent distribution of native species, altitude, flowering time, and life cycle among Delphinieae tribe, *Aconitum, Delphinium, Gymnaconitum* and *Staphisagria* adapted from Jabbour and Renner (2012b). Symbol indicates *exclusive pollination, ^+^introduced species. Notes: *Consolida pubescens* (DC.) Soó¹, *Consolida mauritanica (Coss.) Munz*², *Consolida ajacis* L.³, *A. gymnandrum*ª, *Delphinium staphisagria* L.^b^, *Delphinium picta*^c^. Location data was obtained from the Royal Botanic Gardens Kew website. FNA, Floral of North America; PFAF, Plants for a Future.

| Delphinieae Species & Related Genera | Pollinator Classification | | | | Continent | Altitude (m, asl) | Flowering  (month(s)) | Life Cycle | References |
| --- | --- | --- | --- | --- | --- | --- | --- | --- | --- |
|  | HYMENOPTERA | LEPIDOPTERA | DIPTERA | APODI FORMES | Africa - Eursaia - NorthAmerica |  | | A- Annual  FAB-Facultative Annual or Biennial  P- Perennial  PA-Pseudo-annual |  |
| Aconitum |  | | | | | | | | |
| *A. anthora* |  |  |  |  | E | 1750-1980 | 8-9 | PA | [Royal Botanic Gardens Kew](https://powo.science.kew.org/taxon/urn:lsid:ipni.org:names:707158-1)  (Jabbour and Renner, 2012a, 2012b)  (Bosch et al. 1997) |
| *A. japonicum* |  |  |  |  | E | 500-1500 | 8-10 | P | [Royal Botanic Gardens Kew](https://powo.science.kew.org/taxon/urn:lsid:ipni.org:names:707472-1)  (Jabbour and Renner, 2012b)  (Fukuda et al., 2001)  [Plants for a Future](https://pfaf.org/user/Plant.aspx?LatinName=Aconitum+japonicum) |
| *A. lycoctonum* |  |  |  |  | E | 1600-1980 | 7-8 | P | [Royal Botanic Gardens Kew](https://powo.science.kew.org/taxon/urn:lsid:ipni.org:names:707564-1)  (Jabbour and Renner, 2012a, 2012b)  (Bosch et al. 1997) |
| *A. napellus* |  |  |  |  | E -NA^+^ | 1980-2350 | 7-8 | PA | [Royal Botanic Gardens Kew](https://powo.science.kew.org/taxon/urn:lsid:ipni.org:names:707615-1)  (Jabbour and Renner, 2012a, 2012b)  (Bosch et al. 1997) |
| *A. septentrionale* |  |  |  |  | E | ~500-1000 | - | P | [Royal Botanic Gardens Kew](https://powo.science.kew.org/taxon/urn:lsid:ipni.org:names:707807-1)  (Jabbour and Renner, 2012a, 2012b)  (Fourcade et al., 2019) |
| *A.*  *kusnezoffii* |  |  |  |  | E | 1000-1400 | 7-9 | PA | [Royal Botanic Gardens Kew](https://powo.science.kew.org/taxon/urn:lsid:ipni.org:names:707505-1)  (Jabbour and Renner, 2012a, 2012b)  (Liao et al., 2009)  (Tian et al., 2022) |
| *A. delphiniifolium* |  |  |  |  | E - NA | 0-1700 | 6-9 | PA | [Royal Botanic Gardens Kew](https://powo.science.kew.org/taxon/urn:lsid:ipni.org:names:707308-1)  (Jabbour and Renner, 2012a, 2012b)  [Flora of North America](http://floranorthamerica.org/Aconitum_delphiniifolium) |
| *A. columbianum* |  |  |  |  | NA | 300-3500 | 7-9 | PA | [Royal Botanic Gardens Kew](https://powo.science.kew.org/taxon/urn:lsid:ipni.org:names:707271-1)  (Jabbour and Renner, 2012a, 2012b)  [Flora of North America](http://floranorthamerica.org/Aconitum_columbianum_subsp._columbianum) |
| *A. noveboracense* |  |  |  |  | NA | 400-1000 | 6-8 | P | [Royal Botanic Gardens Kew](https://powo.science.kew.org/taxon/urn:lsid:ipni.org:names:3405-2)  (Jabbour and Renner, 2012b)  [New York Natural Heritage Program](https://guides.nynhp.org/northern-monkshood/)  (Kuchenreuther, 1996) |
| Delphinium |  | | | | | | | | |
| *D. balansae* |  |  |  |  | A | 2400-2800 | 6-7 | P | [Royal Botanic Gardens Kew](https://powo.science.kew.org/taxon/urn:lsid:ipni.org:names:710422-1)  (Jabbour and Renner, 2012b)  (Bosch et al. 1997) |
| *D. dasycaulon* |  |  |  |  | A | 1400-3000 | 2-6 | P | [Royal Botanic Gardens Kew](https://powo.science.kew.org/taxon/urn:lsid:ipni.org:names:710544-1)  (Jabbour and Renner, 2012b)  [Flora of Mozambique](https://www.mozambiqueflora.com/speciesdata/species.php?species_id=170690) |
| *D. favargeri* |  |  |  |  | A | ~1400 | 6-7 | A | [Royal Botanic Gardens Kew](https://powo.science.kew.org/taxon/urn:lsid:ipni.org:names:997334-1)  (Jabbour and Renner, 2012a, 2012b)  (Bosch et al. 1997) |
| *D. leroyi* |  | * |  |  | A | 1500-3500 | - | P | [Royal Botanic Gardens Kew](https://powo.science.kew.org/taxon/urn:lsid:ipni.org:names:710747-1)  (Jabbour and Renner, 2012a, 2012b) |
| *D. pubescens^1^* |  |  |  |  | A-E | 70-1000 | 4-7 | A | [Royal Botanic Gardens Kew](https://powo.science.kew.org/taxon/urn:lsid:ipni.org:names:710934-1)  (Jabbour and Renner, 2012b)  (Bosch et al. 1997)  [IPFI](https://www.actaplantarum.org/flora/flora_info.php?id=2246&nnn=Delphinium%20consolida%20pubescens)  [Flora of North America](http://www.efloras.org/florataxon.aspx?flora_id=1&taxon_id=233500421)  [Herbari Virtual del Mediterranean Occidental](http://herbarivirtual.uib.es/ca/general/1966/especie/consolida-pubescens-dc-soo) |
| *D. mauritanicum^2^* |  |  |  |  | A - E | 1400-2100 | 6-7 | A | [Royal Botanic Gardens Kew](https://powo.science.kew.org/taxon/urn:lsid:ipni.org:names:710791-1)  (Jabbour and Renner, 2012a, 2012b)  (Bosch et al. 1997) |
| *D. ajacis^3^* |  |  |  |  | E- NA^+^-A^+^ | 200-3000 | 6-7 | A | [Royal Botanic Gardens Kew](https://powo.science.kew.org/taxon/urn:lsid:ipni.org:names:77254-2)  (Jabbour and Renner, 2012a, 2012b)  (Bosch et al. 1997) |
| *D. bolosii* |  |  |  |  | E | 290-600 | 6 | P | [Royal Botanic Gardens Kew](https://powo.science.kew.org/taxon/urn:lsid:ipni.org:names:901887-1)  (Jabbour and Renner, 2012b)  (Bosch et al. 1997)  (Bosch et al., 2006) |
| *D. consolida* |  |  |  |  | E | 0-1000 | 6-7 | FAB | [Royal Botanic Gardens Kew](https://powo.science.kew.org/taxon/urn:lsid:ipni.org:names:306669-2)  (Jabbour and Renner, 2012b)  [USDA - Natural Resources Conservation Services](https://plants.usda.gov/home/plantProfile?symbol=CORE2)  [Plants for a Future](https://pfaf.org/user/Plant.aspx?LatinName=delphinium+consolida) |
| *D. gracile* |  |  |  |  | E | <1200 | 7-8 | A | [Royal Botanic Gardens Kew](https://powo.science.kew.org/taxon/urn:lsid:ipni.org:names:710647-1)  (Jabbour and Renner, 2012a, 2012b)  (Bosch et al. 1997) |
| *D. montanum* |  |  |  |  | E | ~2000 | 7-8 | P | [Royal Botanic Gardens Kew](https://powo.science.kew.org/taxon/urn:lsid:ipni.org:names:710815-1)  (Jabbour and Renner, 2012a, 2012b)  (Bosch et al. 1997) |
| *D. sclerocladum* |  |  |  |  | E | ~900-1800 | 5-8 | A | [Royal Botanic Gardens Kew](https://powo.science.kew.org/taxon/urn:lsid:ipni.org:names:710995-1)  (Jabbour and Renner, 2012b)  [Flora of Israel and Adjacent Areas](https://flora.org.il/en/plants/conscl/)  (Munz, 1967) |
| *D. stenocarpum* |  |  |  |  | E | 900-1300 | 6-8 | A | [Royal Botanic Gardens Kew](https://powo.science.kew.org/taxon/urn:lsid:ipni.org:names:711036-1)  (Jabbour and Renner, 2012b)  (Munz, 1967)  [Turkish Plant Data Service](http://194.27.225.161/yasin/tubives/index.php?sayfa=1&tax_id=192) |
| *D. verdunense* |  |  |  |  | E | 200-1500 | 7-8 | A | [Royal Botanic Gardens Kew](https://powo.science.kew.org/taxon/urn:lsid:ipni.org:names:711123-1)  (Jabbour and Renner, 2012b)  (Bosch et al. 1997) |
| *D. barbeyi* |  |  |  |  | NA | 2500-4100 | 6-8 | P | [Royal Botanic Gardens Kew](https://powo.science.kew.org/taxon/urn:lsid:ipni.org:names:77282-2)  (Jabbour and Renner, 2012a, 2012b)  [Lady Bird Johnson Wildflower Center - The University of Texas at Austin](https://www.wildflower.org/plants/result.php?id_plant=DEBA2) |
| *D. bicolor* |  |  |  |  | NA | 70-3100 | 5-7 | P | [Royal Botanic Gardens Kew](https://powo.science.kew.org/taxon/urn:lsid:ipni.org:names:710434-1)  (Jabbour and Renner, 2012a, 2012b)  [Lady Bird Johnson Wildflower Center - The University of Texas at Austin](https://www.wildflower.org/plants/result.php?id_plant=DEBI) |
| *D. cardinale* |  |  |  | * | NA | 50-1500 | 3-7 | P | [Royal Botanic Gardens Kew](https://powo.science.kew.org/taxon/urn:lsid:ipni.org:names:275693-2)  (Jabbour and Renner, 2012a, 2012b)  [Lady Bird Johnson Wildflower Center - The University of Texas at Austin](https://www.wildflower.org/plants/result.php?id_plant=DECA2) |
| *D. decorum* |  |  |  |  | NA | 0-2300 | 3-7 | P | [Royal Botanic Gardens Kew](https://powo.science.kew.org/taxon/urn:lsid:ipni.org:names:30033419-2)  (Jabbour and Renner, 2012a, 2012b)  [Lady Bird Johnson Wildflower Center - The University of Texas at Austin](https://www.wildflower.org/plants/result.php?id_plant=DEDED) |
| *D. glaucum* |  |  |  |  | NA | 0-3200 | 6-9 | P | [Royal Botanic Gardens Kew](https://powo.science.kew.org/taxon/urn:lsid:ipni.org:names:275700-2)  (Jabbour and Renner, 2012b)  [Lady Bird Johnson Wildflower Center - The University of Texas at Austin](https://www.wildflower.org/plants/result.php?id_plant=degl3)  [Flora of North America](http://www.efloras.org/florataxon.aspx?flora_id=1&taxon_id=233500500) |
| *D. nudicaule* |  |  |  | * | NA | 0-2600 | 3-6 | P | [Royal Botanic Gardens Kew](https://powo.science.kew.org/taxon/urn:lsid:ipni.org:names:77433-2)  (Jabbour and Renner, 2012a, 2012b)  [Lady Bird Johnson Wildflower Center - The University of Texas at Austin](https://www.wildflower.org/plants/result.php?id_plant=DENU) |
| *D. nuttallianum* |  |  |  |  | NA | 300-3500 | 3-7 | P | [Royal Botanic Gardens Kew](https://powo.science.kew.org/taxon/urn:lsid:ipni.org:names:77426-2)  (Jabbour and Renner, 2012a, 2012b)  [Lady Bird Johnson Wildflower Center - The University of Texas at Austin](https://www.wildflower.org/plants/result.php?id_plant=DENU2) |
| *D. parryi* |  |  |  |  | NA | 0-1700 | 3-6 | P | [Royal Botanic Gardens Kew](https://powo.science.kew.org/taxon/urn:lsid:ipni.org:names:275714-2)  (Jabbour and Renner, 2012a, 2012b)  [Lady Bird Johnson Wildflower Center - The University of Texas at Austin](https://www.wildflower.org/plants/result.php?id_plant=DEPA2) |
| *D. tricorne* |  |  |  |  | NA | 0-2300 | 3-5 | P | [Royal Botanic Gardens Kew](https://powo.science.kew.org/taxon/urn:lsid:ipni.org:names:711086-1)  (Jabbour and Renner, 2012a, 2012b)  [Lady Bird Wildflower Center -The University of Texas at Austin](https://www.wildflower.org/plants/result.php?id_plant=DETR) |
| *D. virescens* |  |  |  |  | NA | 250-2000 | 3-7 | P | [Royal Botanic Gardens Kew](https://powo.science.kew.org/taxon/urn:lsid:ipni.org:names:77546-2)  (Jabbour and Renner, 2012b)  [Lady Bird Johnson Wildflower Center - The University of Texas at Austin](https://www.wildflower.org/plants/result.php?id_plant=DECAV2)  [Flora of North America](http://floranorthamerica.org/Delphinium_carolinianum_subsp._virescens) |
| *Gymnoaconitum* |  | | | | | | | | |
| *G. gymnandrum^a^* |  |  |  |  | E | 500-4430 | 6-8 | FAB | [Royal Botanic Gardens Kew](https://powo.science.kew.org/taxon/urn:lsid:ipni.org:names:707407-1)  (Jabbour and Renner, 2012a)  (Wang et al., 2009)  [Flora of China](http://www.efloras.org/florataxon.aspx?flora_id=2&taxon_id=200007199)  (Wang et al., 2013) |
| Staphisagria |  | | | | | | | | |
| *S. macrosperma^b^* |  |  |  |  | A- E | 70-160 | 5-6 | FAB | [Royal Botanic Gardens Kew](https://powo.science.kew.org/taxon/urn:lsid:ipni.org:names:60456584-2)  (Jabbour and Renner, 2012a, 2012b)  (Bosch et al. 1997) |
| *S. picta^c^* |  |  |  |  | E | 0-100 | 6 | FAB | [Royal Botanic Gardens Kew](https://powo.science.kew.org/taxon/urn:lsid:ipni.org:names:77115896-1)  (Jabbour and Renner, 2012a, 2012b) |

**Supplementary Table 4:** Expression of MADS-box and symmetry genes at five developmental stages (S1-S2, S4, S6, S9, and S12) in *D*. *ajacis* based on mRNA *in situ* hybridization and DGE profiling results obtained by Zhao et al. (2023). The number of plus signs approximately indicate the level of expression, with three pluses showing higher expression. The minus sign indicates very little to no expression. S, Sepal; P, petal; St, stamen; C, carpel; RP, reduced petal; sSS, single-spurred sepal; NS, non-spurred sepal; sSP, single-spurred petal; D, dorsal; V, ventral.

|  | S1-S2 | S4 | | S6 | | | | | S9 | | | | | | S12 | | | | |
| --- | --- | --- | --- | --- | --- | --- | --- | --- | --- | --- | --- | --- | --- | --- | --- | --- | --- | --- | --- |
|  | FM | S (D&V) | P (D&V) | S (D&V) | P(D) | St | C | RP (V) | sSS (D) | P (D) | St | C | RP (V) | NS (V) | sSS (D) | sSP (D) | St | C | NS (V) |
| *DeajAP3-1* | - | - | ++ | - | +++ | +++ | + | +++ | - | + | +++ | + | + | - | - | - | +++ | - | - |
| *DeajAP3-2* | - | - | - | - | - | +++ | + | - | - | + | +++ | + | + | - | + | + | +++ | + | + |
| *DeajAP3-3* | - | - | +++ | - | +++ | +++ | +++ | - | - | +++ | - | - | - | - | - | +++ | - | - | - |
| *DeajPI1* | - | +++ | +++ | +++ | +++ | +++ | +++ | +++ | +++ | +++ | +++ | +++ | +++ | +++ | +++ | +++ | +++ | - | +++ |
| *DeajPI2* | - | + | + | + | ++ | ++ | + | ++ | - | - | ++ | + | - | - | - | - | +++ | - | - |
| *DeajDIV1* | + | +++ | +++ | + | ++ | ++ | ++ | ++ | +++ | +++ | ++ | ++ | +++ | +++ | +++ | +++ | ++ | +++ | +++ |
| *DeajDIV2a* | + | +++ | +++ | + | ++ | ++ | ++ | ++ | + | +++ | ++ | ++ | +++ | + | + | ++ | + | ++ | + |
| *DeajDIV2b* | - | - | - | + | - | - | - | - | + | + | + | + | + | + | + | + | + | + | + |
| *DeajCYC1a* | - | - | - | - | - | - | - | - | - | - | - | - | - | - | - | - | - | - | - |
| *DeajCYC2a* | - | - | - | - | - | - | - | - | - | - | - | - | - | - | - | - | - | - | - |
| *DeajCYC2b* | +++ | +++ | +++ | + | +++ | +++ | +++ | +++ | ++ | +++ | - | - | - | - | + | ++ | - | - | - |
| *DeajAGL6-1a* | +++ | +++ | +++ | +++ | +++ | + | + | +++ | +++ | +++ | - | + | +++ | +++ | +++ | +++ | - | ++ | +++ |
| *DeajAGL6-1b* | ++ | + | +++ | ++ | ++ | + | + | ++ | ++ | ++ | + | - | ++ | ++ | +++ | +++ | + | + | +++ |
| *DeajAGL6-2* | ++ | + | ++ | + | + | + | + | + | + | + | + | - | + | + | + | + | - | - | + |

**References Cited**

*Aconitum gymnandrum in Flora of China @ efloras.org* (no date). Available at: <http://www.efloras.org/florataxon.aspx?flora_id=2&taxon_id=200007199> (Accessed: January 2024).

Bosch, M., Molero, J., Rovira, A., Simon, J., López-Pujol, J., Orellana, M. R., et al. (2006). Recovery plans for Delphinium bolosii and Thymus loscosii: results from three-year studies and conclusions.

Bosch, M., López-Pujol, J., Blanché, C., and Simon, J. (2023). DCDB: Chromosome Database of Tribe Delphinieae (Ranunculaceae): Structure, Exploitation, and Recent Development. *Methods Mol Biol* 2703, 173–192. doi: [10.1007/978-1-0716-3389-2_13](https://doi.org/10.1007/978-1-0716-3389-2_13)

Bosch, M., Simon, J., Blanche, C., and Molero, J. (1997). Pollination ecology in tribe Delphinieae (Ranunculaceae) in W Mediterranean area: floral visitors and pollinator behaviour. *Lagascalia* 19, 545–562.

Bosch, M., Simon J., López-Pujol J., and Blanché C. (2022 onwards). DCDB: *Delphinieae Chromosome Database* v. 3.1. (last updated: 11.1.2024) Available at: [http://www.delphinieae.online](http://www.delphinieae.online/) Accessed: July 2024).

*Consolida pubescens (DC.) Soó* (no date) *Herbari Virtual del Mediterrani Occidental*. Available at: <http://herbarivirtual.uib.es/ca/general/1966/especie/consolida-pubescens-dc-soo> (Accessed: January 2024).

*Consolida scleroclada (Boiss.) Schroedinger | Flora of Israel and adjuscent areas* (no date) *צמחיית ישראל וסביבתה*. Available at: <https://flora.org.il:443/en/plants/conscl/> (Accessed: January 2024).

*Consolida stenocarpa | Euro+Med-Plantbase* (no date). Available at: <https://www.europlusmed.org/cdm_dataportal/taxon/db6463b7-f2fc-4715-bd1c-13013a100ea2> (Accessed: January 2024).

*Flora of Mozambique: Species information: Delphinium dasycaulon* (no date). Available at: <https://www.mozambiqueflora.com/speciesdata/species.php?species_id=170690> (Accessed: January 2024).

*FNA* (no date). Available at: <http://floranorthamerica.org/Main_Page> (Accessed: January 2024).

Fourcade, Y., Åström, S., and Öckinger, E. (2019). Climate and land-cover change alter bumblebee species richness and community composition in subalpine areas. *Biodiversity and Conservation* 28, 639–653. doi: [10.1007/s10531-018-1680-1](https://doi.org/10.1007/s10531-018-1680-1).

Fukuda, Y., Suzuki, K., and Murata, J. (2001). The function of each sepal in pollinator behavior and effective pollination in *Aconitum japonicum* var. *montanum*. *Plant Species Biology* 16, 151–157. doi: [10.1046/j.1442-1984.2001.00059.x](https://doi.org/10.1046/j.1442-1984.2001.00059.x).

*IPFI sheet, Acta Plantarum Delphinium_pubescens* (no date). Available at: <https://www.actaplantarum.org/flora/flora_info.php?id=2246&nnn=Delphinium%20consolida%20pubescens> (Accessed: January 2024).

Jabbour, F., and Renner, S. S. (2012a). A phylogeny of Delphinieae (Ranunculaceae) shows that Aconitum is nested within Delphinium and that Late Miocene transitions to long life cycles in the Himalayas and Southwest China coincide with bursts in diversification. *Molecular Phylogenetics and Evolution* 62, 928–942. doi: [10.1016/j.ympev.2011.12.005](https://doi.org/10.1016/j.ympev.2011.12.005).

Jabbour, F., and Renner, S. S. (2012b). Spurs in a Spur: Perianth Evolution in the Delphinieae552(Ranunculaceae).International Journal of Plant Sciences173, 1036–1054. doi:10.1086/667613.

Kuchenreuther, M. A. (1996). The Natural History of Aconitum noveboracense Gray (Northern Monkshood), a Federally Threatened Species. *Journal of the Iowa Academy of Science: JIAS* 103, 57–62.

*Lady Bird Johnson Wildflower Center - The University of Texas at Austin* (no date). Available at: <https://www.wildflower.org/plants/> (Accessed: January 2024).

Liao, W.-J., Hu, Y., Zhu, B.-R., Zhao, X.-Q., Zeng, Y.-F., and Zhang, D.-Y. (2009). Female reproductive success decreases with display size in monkshood, Aconitum kusnezoffii (Ranunculaceae). *Annals of Botany* 104, 1405–1412. doi: [10.1093/aob/mcp237](https://doi.org/10.1093/aob/mcp237).

Luo, X.-Y., Nie, T.-J., Liu, H., Ding, X.-F., Huang, Y., Guo, C.-C., et al. (2023). Karyotype and genome size variation in Delphinium subg. Anthriscifolium (Ranunculaceae). *PK* 234, 145–165. doi: [10.3897/phytokeys.234.108841](https://doi.org/10.3897/phytokeys.234.108841)

Munz, P. A. (1967). A Synopsis of the Asian Species of Consolida (Ranunculaceae). *Arnold Arboretum of Harvard University* 48, 159–202.

*Northern Monkshood Guide - New York Natural Heritage Program* (no date). Available at: <https://guides.nynhp.org/northern-monkshood/> (Accessed: January 2024).

*PFAF* (no date). Available at: <https://pfaf.org/user/Default.aspx> (Accessed: January 2024).

*Plants of the World Online | Kew Science* (no date) *Plants of the World Online*. Available at: <https://powo.science.kew.org/> (Accessed: January 2024).

Tian, H., Harder, L. D., Wang, A., Zhang, D., and Liao, W. (2022). Habitat effects on reproductive phenotype, pollinator behavior, fecundity, and mating outcomes of a bumble bee–pollinated herb. *American J of Botany* 109, 470–485. doi: [10.1002/ajb2.1826](https://doi.org/10.1002/ajb2.1826).

*TUBIVES Consolida stenocarpa* (no date). Available at: <http://194.27.225.161/yasin/tubives/index.php?sayfa=1&tax_id=192> (Accessed: January 2024).

*USDA Plants Database* (no date). Available at: <https://plants.usda.gov/home/plantProfile?symbol=CORE2> (Accessed: January 2024).

Wang, W., Liu, Y., Yu, S.-X., Gao, T.-G., and Chen, Z.-D. (2013). Gymnaconitum, a new genus of Ranunculaceae endemic to the Qinghai–Tibetan Plateau. *TAXON* 62, 713–722. doi: [10.12705/624.10](https://doi.org/10.12705/624.10)

Wang, L., Abbott, R. J., Zheng, W., Chen, P., Wang, Y., and Liu, J. (2009). History and evolution of alpine plants endemic to the Qinghai‐Tibetan Plateau: *Aconitum gymnandrum* (Ranunculaceae). *Molecular Ecology* 18, 709–721. doi: [10.1111/j.1365-294X.2008.04055.x](https://doi.org/10.1111/j.1365-294X.2008.04055.x).

Zhao, H., Liao, H., Li, S., Zhang, R., Dai, J., Ma, P., et al. (2023). Delphinieae flowers originated from the rewiring of interactions between duplicated and diversified floral organ identity and symmetry genes. *The Plant Cell* 35, 994–1012. doi: [10.1093/plcell/koac368](https://doi.org/10.1093/plcell/koac368)
